# Redefining ‘Worlds’: the writerly ethics in the trailing temporality of J. M. Coetzee’s *Disgrace*

**DOI:** 10.1186/2193-1801-2-143

**Published:** 2013-04-04

**Authors:** Narasimha Rao Kedari

**Affiliations:** Department of English, Vikrama Simhapuri University, Nellore, 524 003 Andhra Pradesh India

## Abstract

South Africa going to second democratic elections saw two path-breaking works exploring disgraceful crimes and emotional commotions in an intriguing era. One is J.M. Coetzee’s *Disgrace* (1999) and Andre Brink’s *The Rights of Desire* (2000). The Protagonists of both the works are middle-aged caught in the noose of *Eros* and are seen resisting the change of tides and resigned to the fateful happenings in store for them. The novel portrays the transitional apprehensions of the whites, the power-wielders of the yester- years to adapt to the syndrome of power withdrawal and deprivation. The story depicts individual self-denigration in a changed political environment dictating a code of moral uprightness and ethics. The redeeming consolation of comic, grotesque and lunatic overtures that Beckett ingeniously provides in his fiction do not find a place in Coetzee. Instead Coetzee forces his readers to look into ineluctable gaps that mark the narration.

In the late 1990s, Coetzee is at work on another compelling novel set in South Africa. The struggle against the repressive, racist state is finally over, apartheid is a discredited policy of the past, and democratic government has finally been established. The age of iron is no more. Has South Africa re-entered at last one of those “softer ages” longed for by Mrs. Curren in her reinvention of Hesiod’s creation narrative? The new novel, *Disgrace*, published in 1999, certainly suggests that the ten or twelve years that have passed since Mrs. Curren’s dying days have indeed wrought a transformation in the country, but it’s not easy to say what age we find ourselves in now. A time of rampant crime, inefficient police services, middle-classes barricaded into their fortress-homes: have we followed Mrs. Curren’s inverted sequence and moved beyond iron only to reach bronze? “In this place, at this time” Coetzee’s fiction has always had a mixed reception in South Africa, and it’s very success elsewhere in the world has increased the suspicion felt among some groups in his native country.

Coetzee’s *Age of Iron* (1990), the novel that appeared before *Disgrace* (1999b), does engage history by attempting to create the conditions that are necessary for the ethical to meditate on the political circumstances. Inventing a new mode of narrative and discovering a new syntax every time, Coetzee in the succeeding novel, *The Master of Petersburg* (1994) peers into the abyss of revolution, chooses the plunge into writing, and mocks the romantic-apocalyptic connotations conventionally associated with both those choices. *Disgrace* (1999b) has a clear thematic connection as Coetzee explores in *The Master of Petersburg*, the protagonist, learning to love by humbling himself and by coming to terms with violence and death. In *Disgrace*, this occurs both through a tragic personal encounter with violence and through David’s volunteer work at an animal clinic. The novel also presents the national public spectacle of shame, confession, and forgiveness that was the *Truth and Reconciliation Commission*, problematizing notions of morality and engaging with Dostoevskyan skepticism. The struggle against the repressive, racist state is finally over as apartheid is a debris heaped upon which the democratic government has been established.

South Africa going to second democratic elections saw two path-breaking works exploring disgraceful crimes and emotional commotions in an intriguing era. One is J.M. Coetzee’s *Disgrace* (1999b) and Andre Brink’s *The Rights of Desire* (2000). The Protagonists of both the works are middle-aged caught in the noose of *Eros* and are seen resisting the change of tides and resigned to the fateful happenings in store for them. David Lurie’s romantic leap with a student is “a last leap of the flame of sense before it goes out” (27). *Disgrace* presents a study of Individual’s closely guarded quarters encroached upon by the political outcomes. It explores the conflict between desire and love, and public disgrace and individual grace.

It portrays the transitional apprehensions of the whites, the power-wielders of the yester- years to adapt to the syndrome of power withdrawal and deprivation. The story depicts individual self-denigration in a changed political environment dictating a code of moral uprightness and ethics. It delineates the tragic outcomes of sexual deviance enslaved by sensual indulgences and imposing sexual brutality as an invincible weapon for taking control of a hapless woman who resolves to make her own life thereby enlivening the relations and confrontations of the communities dictated by history.

In the novel’s opening movement, the protagonist, David Lurie, loses the cautious balance he has maintained, under the new regime, as a Professor in the Communications Department of a large university. Disgracing himself through an affair with a female student, Melanie Issacs, Lurie loses his job and finds himself adrift in a society variously hostile, inscrutable and unpredictable South Africa. Lurie is broadly representative of an older social order and the officially defunct South Africa of Afrikaner dominance, statutory racial oppression and the uneasy pleasures of the white privilege. A sort of truncated Bildungsroman, *Disgrace* demonstrates Lurie’s confrontation with change. This confrontation is precipitated early through the text’s striking of a note of complacent equipoise, followed by loss and a contrasting note of desperation. Lurie finds “entirely satisfactory” his weekly arrangement with the prostitute Soraya. His Thursday interludes of “*luxe et volupte*” (1) in Green Point flat offer him the combination of sensuality and utilitarian order that his ‘temperament’ requires. Sex, for Lurie, is somewhat theoretical - a problem that he feels has solved “rather well” (1). His uxorious but uncommitted feelings for Soraya are disturbed, when he sees her shopping in town with her sons. The unpredictably of the quotidian breaks into their enclave and she refuses to see him again. Thrown off balance, Lurie exists for a while “in an anxious flurry of promiscuity” (7). His amorous powers suddenly departed, he albeit stalks the enigmatic Melanie and their affair further perturbs his sexual confidence. Expelled from the University after Melanie files a complaint against him, Lurie finds his assumptions about sex – as controllable and governed basically by the principles of the hunt – challenged.

After the seduction of Melanie has come to an unholy end, Lurie, now the unceremonious figure of an official harassment enquiry dines with his former wife. She curtly tells David,

Don’t expect sympathy from me, David,” she warns him, “and don’t expect sympathy from anyone else either. No sympathy, no mercy, not in his day and days.” (44)

Perhaps these lines echo the process of dehumanization and the demands of rationalization. Coetzee articulates the change of times through sexuality which becomes a kind of flexible but ambiguous trope for the wider historical changes he registers. Forced to resign from the university, Lurie seeks refuge with his daughter Lucy on a smallholding in the Eastern Province, where she grows flowers and vegetables for the market in nearby Grahmstown and runs dog kennels. Here Lurie meets Petrus, the African who assists Lucy and who along with Lucy owns the property. Petrus shares his concern with David about Lucy’s isolated life. “it is dangerous,… . Everything is dangerous today,” (64). The times reflect not just those who are bare of privileges but who in true sense experience the deprivation as such. Petrus, however, remains almost entirely inscrutable, and merely could be giving polite assent to Lurie’s comment; as so often in Coetzee’s fiction, the racially or socially privileged character can gain virtually no understanding of the inner world of the other who has been excluded from such privilege.

Lurie is given the chance of getting a reprieve from the disgraceful act on his complying with the committee’s demands of the probe into Melanie’s issue accepting and confessing in the public and his preparedness for the counseling. Lurie is resistant to these demands since he is averse to the newly-asserted institutional rights and newly-emergent collective mores that relegate him to submission. “These are puritanical times,” he says, “Private life is public business,” (66). He explains to Lucy the he can’t mount a public defense of his actions, “The case you want me to make is a case that no longer be made *basta*, not in our day.” (89). This shift is not a consequence of the apartheid but the dynamics happening tossed in the global milieu. The disciplinary committee charged with punishing Lurie for his sexual affair requires him to publicly concede in a confessional statement that triggers polarizing resonances. The disciplinary committee attaches the label of human rights violation to Lurie’s affair which falls in line the Truth and Reconciliation Commission’s (TRC) resolve to amplify standard categorization of human rights offenses to include crimes that were not covered by former truth commission. TRC has broadened its definition by condemning “severe ill treatment” apart from “killings, disappearances and tortures” that is stated by Andrew Rigby (2001) in *Justice and Reconciliation: After the Violence*. He further declares – a potentially vague and amorphous description that unsurprisingly spawned heated criticism within South Africa (90) that can also be found in Michael Ignatieff’s (2001) “Human Rights as Politics and Idolatory.” Lurie’s own assault and his vicarious experience of violation through Lucy’s rape instigate Lurie’s reversal in his attitudes toward the law. In lieu of his earlier disparagement of legal redress, after becoming the object of crime, Lurie seeks not only retribution but also the symbolic verification offered by the law. Lurie’s unstable behavior stages a quasi-theoretical debate about law’s abiding to its framed ‘principles’ in an emerging social and political transformation. In a recent essay on the intellectual in South Africa, Coetzee (1999a) in “Critic & Citizen: A Response” muses on

… . . a process of intellectual colonization going on today that… . originates in the culture factories of the United States, and can be detected in the most intimate corners of our lives, or if not in our own then in our students’ lives. (111)

Much of the early section of the novel reads like a satire that is unlikely of Coetzee poised to claim relevance with his situation as Professor of Literature in the University of Cape Town. The mood begins to shift and becomes solemn when Lurie reaches the Eastern Cape to his daughter’s small land holding. It initially appears as a retreat from the complexities of life in post-colonial South Africa. Though separated from the increasingly urban life of contemporary South Africa, the countryside forms a theoretical blank slate upon which a new culture is to be formed. The action that happens on Lucy’s tiny strip of land is thus the drama of South African future. It gets transformed when one day the strip of land is attacked by two men and a boy, all black. They shot down the dogs in the kennels, burnt Lurie and stole his car and the worst of all Lucy is gang-raped.

This event turns the novel more solemn and darker. Lurie’s shock affects his sense of being possessed by angry sarcasm that reflects in his changed moral views and his perception of social landscape. This changed phenomenon which calls for the propriety of instantly attributing the events to the author’s dissolution is also not completely dispossessed of the direct references in the changed political scenario. A risk that one owns of possessing anything,

… . a car, a pair of shoes, a packet of cigarettes. Not enough to go around, not enough cars, shoes, cigarettes. Too many people, too few things. What there is must go into circulation, so that everyone can have a chance to be happy for a day… . That is how one must see life in this country: in its schematic aspect. Otherwise one would go mad. Cars, shoes; women too. There must be some niche in the system for women and what happens to them. (98)

It is in the aftermath of this attack Coetzee’s examination of whiteness gains a caesura in history as a social identity in South Africa. In the aftermath of the fire that ravages Lurie’s face and head, Coetzee writes in *Disgrace*,

Save for a patch over one ear, he seems to have no hair, his whole scalp is tender. Everything is tender, Everything is burned. Burned, burnt (97)

The fire that marks Coetzee’s most straightforward attack on the character he criticizes throughout also announces the passage into a new kind of ‘post-ness’, to borrow the term Atwell uses to describe the effect of the perfective, one in which Lurie will face an irreversible decline. The three intruders being black and shown in negative light has become the object of controversy. Lurie is a typical white South African that grew up with apartheid – he was just born after three years after Nationalist Government won power. He possesses the liberal views claiming of his intellectual allegiance to the English speaking white population. He is restless to bring the culprits to law falling oblivious to his own sexual crime. Lucy, in contrast has a different attitude not entertaining any initiative to bring charges against the man who molested her. She says,

What happened to me is purely private matter. In another time, in another place it might be held to be a public matter. But in this place, at this time, it is not. It is my business, mine alone.” (112)

When Lurie asks “This place being what?” she answers, “This place being South Africa,” (112). Lurie, who views Petrus as the Other suddenly, is moved by the change that happens in Lucy to whom he has been a loving and attentive father. Clinging on to the values and habits of a lifetime, Lucy takes a shocking decision regretting the erosion of values and seeks a new accommodation through her willingness to become Petrus’s third “wife” being fully conscious of the price she is paying. Lucy wants a new shelter, a new accommodation that can guard her. Petrus’s absence during the attack is no coincidence but it appears that he wants Lucy to reduce to a condition of dependency, a by-owner of the farm. Lurie cannot digest the situation, he becomes helpless and just recalls the old days.

In the old days one could have it out with Petrus. In the old days, one could have had it out to the extent of losing one’s temper and sending him packing and hiring someone in his place… . . it is a new world they live in, he and Lucy and Petrus. Petrus know it, and he knows it, and Petrus Knows that he knows it. (116–17)

Lurie’s dissolution with the contemporary political order becomes perceptible in the aftermath of the apartheid. The question of race getting faded away by the dynamics of human relations is so inconsistent and unreliable. Strange to the political triggers of South Africa, the changed times do not reflect the huge strides of technological advancement of the new era but portrays the dark story sliding into fathomless atavism. Lurie, in the grip of inability to control the situation treats the attack as inevitable, the result of a deterministic historical process over which individual human beings can possess no control. Even though the post-historical mood that Coetzee utilizes negates such a sense of history right from the beginning, Lucy thinking of the bad memory tells,

It was so personal. It was done with such personal hatred… . . why did they hate me so? I had never set eyes on them. (156)

The question which seems unrhetorical ably finds a response in Lurie, as he muses,

It was history speaking through them… . A history of wrong. Think of it that way, if it helps. It may have seemed personal, but it wasn’t, It came down from ancestors. (156)

Lurie is struck by the violence unleashed with so much of disgusting hatred. He cannot simply justify that history revisits with retaliating vengeance, an outcome of European colonialism in Africa. The attack redefines the white identity and also reaffirms Lurie’s viewing his African counterparts as essentially barbaric. He is hapless as he assumes himself to be in a savagious place haunted by bloody cannibals besieging his fortunes. He says,

… . Italian and French will not save him here in darkest Africa. He is helpless, an Aunt Sally, a figure from a cartoon, a missionary in cassock and topi waiting with clasped hands and up cast eyes while the savages jaw away in their own lingo preparatory to plunging him into their boiling cauldron. Mission work: what has it left behind, that huge enterprise of upliftment? Nothing that he can see. (95)

Lurie’s discontent becomes explicit as he views South Africa still in colonial terms of being “dark” at the instance of the weight of the tragic occurrences, bringing out the dormant feelings of racism. The change for Lurie is intangible as he is bogged down by the stepping up of violence and his perplexity at the religious zeal of the missionaries drawing bland blankness of emotional transformations. Lurie suspects Petrus as an accomplice in crime to evacuate Lucy from the land she owns. To realize this plan he resorts to the endemic violence characteristic of South Africa, a resort to the genre of tribal foundations of African life. This proves that Petrus and his Xhosa maneuverings cannot escape Lurie’s intellectual control.

Lucy counters her father’s colonial pre-occupations and typical presumptuous since she cannot alienate herself from South African ethos. Regardless of Lucy’s destiny intertwined with South Africa’s future, Lurie plans of going back to Europe asking her daughter for her consent. He pleads,

Close down the kennels. Do it at once. Lock up the house, pay Petrus to guard it. Take a break for six months or a year until things have improved in this country. (157)

The emotional distance of being a South African is perceptible in Lurie which stands no case for Lucy as she denies,

Thank you for the offer, but it won’t work. There is nothing you can suggest that I haven’t been through a hundred times myself. (157)

Lucy is firm in staying back and she never estranged herself from the identity of being a white and a South African. This commingling of identities points to her ably negotiating the post-apartheid, post-colonial and post-historical power structures which to Lurie’s viewpoint stand absurd and abstruse. The fissure between the father and the daughter deepens at Lucy’s resolution to bear the pregnancy and her wishful submission as wife to Petrus in which she devoutly aspire her security. Lucy tells her father

Petrus is not offering me a church wedding followed by a honeymoon on the wild coast. He is offering an alliance, a deal. I contribute the land, in return for which I am allowed to creep under his wing. Otherwise, he wants to remind me. I am without protection, I am fair game. (203)

The narrator drops back in silence to make Lurie’s hopelessness more concrete and responds again with disgusting unease.

More and more she has begun to look like one of those women who shuffle around the corridors of nursing homes whispering to themselves. Why should Petrus bother to negotiate? She cannot last: leave her alone and in due course she will fall like rotten fruit. (203)

Lucy resolving to marry Petrus shows the drastic assimilative moves of the power structures of South Africa whose identity is no longer immune to the racial code but sheerly exists in a state of true hybridity alongside a plethora of post-apartheid identities.

The utterances of Romanticism by J.M. Coetzee are really intriguing and engaging since they point to deeper implications of their presence in the text. Jane Taylor (1999) in “The Impossibility of Ethical Action” refers to “the European Enlightenment’s legacy of the autonomy of the individual” (25), a renowned model of philosophical sympathy. The location of these passages refer to Lurie’s academic interests characteristic of his undeniable white colonialist perspective but unpresumptuous of Coetzee’s literary project of juxtaposing two famous yet explicitly antagonistic Romantic poets. There are criticisms which adopt anti-Eurocentrism adopting a contradictory stance to David Lurie’s Romantic preoccupations. The Ethical and political ramifications of early half of nineteenth century Romanticism cannot just be relegated to mere sophisticated metropolitanism. The South African context is attributed the ideal parallels steamed out by Romanticism throwing challenges at the conventional odds and conservative pretexts, characteristic of the troubled decodes of early 19th century Europe. Romanticism contested the much prompted atomistic tendencies augmented by industrial revolution with its coincidental destruction of the planet, the revival of humaneness is once again given a fresh lease by Coetzee allowing the growth of empathy and being sensitive to the needs of both humans and animals. Despite Lurie’s bearings suggesting his lack of psychological and emotional involvement, it is intriguingly uncertain as to which aspects are vehemently deplored by Wordsworth and Byron.

These Romantic factors draw attention of many well known critics. Michael Marais (20001) in “Very Morbid Phenomena: Liberal Punk, the Lucy-Syndrome and J.M. Coetzee’s *Disgrace*,” talks of Coetzee’s “respect for the otherness of other beings,” a pertinent Wordsworthian concern (38). The Hegelian interest of “the relation of dominance and subservience” (33) is markedly present in Marias critique which further throws light on the narrative being “determined by a tension between desire and responsibility” (174), the imposing preoccupations of the Romantics. Coetzee brings the two contrasting poets together from the High Romantic period negotiating the difficulties underlying human relations of the contemporary world.

Coetzee deftly handles the Romantic interventions of Wordsworth and Byron that spell out dichotomies that affect the structure of the novel. The novel, Disgrace opens up in Cape Town and moves to a farm near Salem, a shift from the urban to the rural. The atmosphere also bears this transformation from the “brisk winter air” (11) “to spring” (196), and finally “the summer season of blooming” (216). Lurie studies Western Romanticism on which he has written three books: one on the “Genesis of Mephistopheles” (via Boito’s *Faust*), one on “Vision as Eros,” the third on “Wordsworth and the Burden of the Past” (4). Devil plays the centre of all these tracts. Satan, a symbol of fallenness, venomous seduction, danger and corruption but also of change growth and spirituality stands as a totem to David Lurie’s sexual temperament is described as “lengthy, absorbed, but rather abstract, rather dry, even at its hottest” (3). Lurie plans of writing an opulent Gluck-like opera, Byron in Italy, which suggests Romantic eroticism through notorious seduction. The novel’s shift in the setting from urban to the rural connotes the overturning influence of French Revolution with the rural taking control of the urban. This urban–rural contest is analogous with the supposedly simple Wordsworth and sophisticated Byron, which complement the novel’s fabric.

Lurie is concerned with his waning sexual passion and his inconsequential interrogation of Melanie is deeply a Byronic concern. Byron created works like Cain, Mazeppa, Don Juan the heroes of which are autobiographically revealing. He, with all his stand alone characteristic features of being libertine, and a rebellious satirist is summed up as a caricature and who in the words Caroline Lamb is “mad, bad and dangerous to know” (77) which Lucy borrows to describe her father. Sexual passion is too important to Byron which is not the case with father. Lurie busies himself with Byron’s *Letters* of 1820 which depict the fleeting nature of passion and its concomitant tragedy. Lurie reading the great lyric of 1817 elicits “The end of roving. Though the heart be still as loving and the moon be still as bright. Who would have thought it would come to an end so soon and so suddenly.” (120) Passion forms a stately subject in Byron, a creative and a destructive force.

Byron’s self exile is related to ‘disgrace’ as Lurie informs the students, “… . notoriety and scandal affected not only Byron’s life but the way in which his poems were received by the public” (31). Wordsworth draws analogy with ‘grace’ the word repetitively occurs in his poems. *Disgrace* draws from the poems of Wordsworth the two female characters, Lucy and Teresa whose actions and bearings form a vital part of the novel’s meaning. Lurie strikes conversation with Melanie about Wordsworth being one of his masters and feels assured “for as long as he can remember the harmonies of *The Prelude* have echoed within him” (15). Lurie quotes from *The Prelude* of Wordsworth contrasting two ways of seeing, seeing *literally* with the eye, and seeing imaginatively. Lurie, through an instrumental use of Wordsworth’s text to convey “covert intimacies” (23) makes advances to Melanie with an utter disregard to his master’s poem. Coetzee draws closer to Wordsworth at the end of chapter Five:

William Wordsworth (1770 – 1850), nature-poet. David Lurie (1945 - ?), commentator upon, and disgraced disciple of, William Wordsworth. Blest be the infant babe. No outcast he. Blest be the Babe.” (46)

Surpassing the empathy that the poet expresses to both animate and inanimate nature is the unbound moral sense that the babe cultivates in responding to the other. It is same identity that Coetzee relies on, at the instance of the dogs, Bev Shaw and Pollux, the difficult path that Lurie is endorsed with effecting an ironic closure to Chapter Five. Lucy might have been named by Lurie after Wordsworth’s Lucy since Lurie keeps his master in the priority of things. The Lucy poems of Wordsworth exclusively touch upon a strange combination of the simplicity of expression and the ambiguity of meaning dealing with issues of Otherness, change and death. Lucy dwells unassumingly as Wordsworthian Lucy in her solitary farm

‘And you? Is this what you want in life? He waves a hand toward the garden, toward the house with sunlight glinting from its roof. ‘It will do,’ replies Lucy quietly. (70)

The imagery of Wordsworth reverberates in Lurie’s dealings as the narrative progresses. Lucy telling her edited version to the police about the incident concealing “Lucy’s secret; his disgrace,” (109). Her partly-true narration omitting her secret rises from her sense of existing with grace, on the other hand Lurie meeting Mr. Issacs speaks of the disgrace befallen to stay with him for long. Lurie is changing, he is “losing himself by day” (121). He initially sees this as negative, “this is not what he came for… . .If he came for anything, it was to gather himself, gather his forces” (121). Later Lurie realizes that even “if he loses himself [he can] be there,” at least imaginatively (160). More than his losing himself, Lurie is more concerned about Lucy’s growing inconceivable. He takes her “living in the shadow of the attack… . .a darker person altogether” (124). This inconceivability, paradoxical nature, and enigmatic disposition have in fact been the essential genres of Wordsworth’s Lucy. Lurie always looks up to his daughter guiding him which has already been happening rather than the reverse. Lurie is not so inventive in comprehending the determination and the intricacy of Lucy’s resolve to stay in the small landholding. He reveals to Issacs “He loves his daughter, but there are times when he wishes she were a simple being: simpler and neater” (170).

Returning to Salem Lurie finds Lucy re-energized. She challenges him to rethink her position in his hierarchy

You behave as if everything I do is part of the story of your life. You are the main character, I am a minor character who doesn’t make an appearance until halfway through. Well, contrary to what you think, people are not divided into major and minor. I am not minor. I have a life of my own, just as important to me as yours is to you, and in my life I am the one who makes the decisions. (198)

Through Lurie, Lucy’s farm house gets a panoramic view as he takes a stance from the hill crest. He watches her as “she bends over, clipping or pruning or tying” (217). This description brings to mind of the famous lines of “The Solitary Reaper.” Lurie develops a vision for the future. “When he is dead she will, with luck, still be here doing her ordinary tasks among the flowerbeds” (217) which informs Lurie’s inimitable acceptance of Lucy’s chosen life. Lurie finds his lack of insight “…. he has never had much of an eye for rural life, despite all his reading in Wordsworth” (218). At last Lurie recognizes Wordsworthian inner eye of imagination as a moral force reinforcing him to give up the dog that he has begun to love, “Are you giving him up?” “Yes, I am giving him up” (220).

Like other novels of Coetzee, *Disgrace* has the allegorical finesse that indicates the black characters un-empathetic to their endorsed individual tragedies. The novel downplays this discord as just the mechanistic response that ignores the novel’s protean dimensions. The novel bounds the pathetic conditions of animals and it becomes intense as Lurie reaches his rural farm where Lucy keeps the dogs for their owners. All these are watch-dogs betokening the conditions in new South Africa about the general state of anxiety about crime. Lurie at first is disturbed by their relentless barking, but he strikes affinity with Katy, a dispossessed bull dog and he likens the dog to the widowed and loverless Teresa Guiccoli. Unguarded of the over-possessing feelings, Lurie falls asleep in Katy’s cage that draws similarity with his sexual reliefs with various women. Lurie acquaints with Bev Shaw, who runs an animal clinic the enterprise of which initially disregards the job. Lurie against this initial reaction offers to help Bev Shaw in her work “feeding, cleaning, mopping up” (142). The changed man cannot see the two sheep tethered in Petrus’s barren farm as he moves them to the grass patches. The change seems unpresumed with Lurie prioritizing the animal lot. His attachment is neither emotional nor unrelenting like the argumentative activist of animal rights. Lurie’s post-Cape Town saga is spent in animal pens reckoning moments of Lucy’s pregnancy and occasionally striking the notes of Teresa’s banjo. As is Coetzee’s vogue not to interfere with his authorial voice to reflect on social events effecting political clout, Lurie’s predicament cannot be taken as his being reclusive to new South African scenario. A novel abiding by the demands of time and place negotiating complex issues of divergent genre draws to an unassuming close landing in an irresolute terrain.

The unassuming close of the novel is not bereft of seriousness, commitment and responsibility as it offers claims for reassurance and utopian moments of social harmony. By way of braiding the narrative with operatic musing and pathetic reflections on animals, Coetzee appears to provide appropriate solutions through giving a call for the production of art and the affirmation of animal lives. Lurie has no illusions about his odd and ludicrous work. He says,

It would have been nice to be returned triumphant to society as the author of an eccentric little chamber opera. But that will not be. (214)

Deeply engrossed in ethical issues that preoccupy his work, desire troubles the narrative readability complementing the philosophical and moral universe of the text in ambivalence. Desire broadens the reach of the ethical by throwing it back onto materiality, reprojecting the space of the body in a phantasmatic light, as both familiar and strange. By these means, it can thus be abstracted from Jacques Derrida’s (1994) work *Specters of Marx: The State of the Debt, the Work of Mourning, and The New International* that desire works as both a reflection of and conduit for “the most intimate stranger… .the *other* within, “whose power inscribes the beyond in the inside, in the essence of the living” (106, 141). In love with the “living body,” desire also wages war against “whatever is not the body but belongs to it, comes back to it: prosthesis and delegation, repetition, *differ*a*nce* (141). Coetzee plays upon the foundering of the intellect and the random abundance of grace depending intimately on the body. The same gets echoed in *Elizabeth Costello* reiterating Coetzee’s profound wish for divine and human conjunction. In the chapter “Eros,” the aging novelist meditates on the myth of Eros and Psyche. “What intrigues her is less the metaphysics than the mechanics, the practicalities of congress across a gap in being” (184). Such prodigal eroticizing of the immortal body, Costello shapes desire as a vehicle for spiritual yearning. She asks: “Can we be one with a god profoundly enough to apprehend to get a sense of a god’s being? (189–90)

Coetzee places the protagonist in an experiential role as he becomes the perpetrator of a rape and engulfed by desperation at the rape of his daughter by the assailants. The writer introduces him to the abject sorrow of being the recipient of crime. This sympathetic imagination bestows on Lurie an empathy for the animals which by the end of the novel develops in him the ability of imaginatively identifying with animals. The novel even in its end continues to manifest subtle shifts in Lurie revealing that he is no longer oblivious to others. He decides not to sleep with prostitutes and to continue working at an animal shelter. The ending focuses on Lurie’s decision to euthanize a dog that dearly loves him. The narrative suggests not just the imaginative experience of Lurie but an image of self-sacrifice that informs Coetzee’s conception of the imagination. Lurie is “bearing him in his arms like a lamb” and states “I am giving him up” (220). Isidore Diala (2001) in “Nadine Gordimer, J.M. Coetzee and Andre Brink: Guilt, Expiation and the Reconciliation Process in Post-Apartheid South Africa,” states “charity and artistic creativity are, of course, in themselves sacrificial and are traditionally regarded as acts of redemption” (58). Elleke Boehmer in (2002) “Not Saying Sorry. Not Speaking Pain: Gender Implications in *Disgrace*” agrees “The surrender of self through empathy is a state which Lurie in time comes to achieve” (346). Coetzee furnishes *Disgrace* with the texture of anti-Bildungsroman, a novel that manifests the dispossession rather than the consolidation of the protagonist’s self. In the process, ethical shift becomes perceptible from Lurie’s assertion his “rights of desire” (52) and describing himself as “Servant of Eros” (88) undergoes a change which finds him in the service of other beings and attending to the daily chores which are unpresumptuous

This is not what he came for – to be stuck in the back of beyond, warding off demons, nursing his daughter, attending to a dying enterprise. If he came for anything, it was to gather himself, gather his forces. Here he is losing himself day by day. (121)

Pamela Cooper (2005) in “Metamorphosis and Sexuality: Reading the Strange Passions of *Disgrace*,” asserts that

… . Body becomes the site of an aesthetic tradition defunct yet tenacious, and of an erotic- of romanticized domination/submission – outdated but clamorous. (31)

Sexuality strikes consonance both with the language of romantic presence and with the hole in the text both empty and pregnant with immense meaning. Lucy Lurie fills this space while engendering its emptiness. Lucy is projected as a lesbian family alone in the absence of her lover (variously named Helen and Grace). The unexpected violence visited upon her creates grievous ripples in her life. The non-phallic sexuality that Lucy chooses is threatened and is “usurped upon,” the phrase Lurie uses in *Disgrace* (22). Lucy is deliberately drawn back into the phallic system where in she consents to marry her “neighbor,” the aspiring land owner Petrus, who symbolizes the patriarchal forces gaining power in post-Apartheid South African society. Lucy is thus abducted from the non-phallic radical sexuality into a neo-masculanist patriarchal society.

Lucy’s conceals from her father what has happened during the attack as he wants to know in full about the incident and is intent on to control the interpretation. Lucy’s refusal brings to light Gayatri Spivak Chakraborthy’s (1991) argument in “Theory in the Margin: Coetzee’s *Foe* Reading Defoe’s *Crusoe/Roxana*,” When there are holes in the narrator’s story, however, it highlights the “fact’ of “mut(e)ilation” (164). Spivak suggests that the subaltern presence may be powerfully grafted in the gaps and omissions of the text.

Rosemary Sorensen in the review of Disgrace puts it that the novel in no way provides easy solutions to “the ethical minefield of South Africa” (7). In “Interview” with David Atwell published in *Doubling the Point: Essays and Interviews*, Coetzee referring to the tension between politics and ethics in his fiction says “I think you will find the contest of interpretations I have sketched here – the political versus the ethical – played out again and again in my novels” (338). Though the interview dates back, Coetzee’s affirmation still holds good. *Disgrace* has all the features that resonate Coetzee’s strikingly negotiating historical surprises. The Writer assumes the responsibility of interrupting the arbitrariness and absoluteness infinitely and these endeavours are fundamentally ateleological. Hence, the new political order of South Africa is also questioned, given the ethical responsibility of Coetzee to continue his interruptive engagement with political totalities in the present. Salman Rushdie (2000) in “Light on Coetzee” suggests that the novelist *colludes* in David’s self-justification sot that the novel “merely become(s) a part of the darkness it describes” (2000:7), though Coetzee is always alert to the possibility of novelistic complicity. In his endorsement of guilt by focusing elsewhere Mike Marais (2000b) in “The Possibility of Ethicla Action: J.M. Coetzee’s *Disgrace*” points out that it is not Coetzee but David Lurie who is “implicated in the instrumentalizing logic which defines relations” in South Africa, so that he is “party to that which he condemns” (58).

Michael Marais (2000a) in the concluding lines of “‘Little Enough, Less than Little: Nothing:’ Ethics, Engagement and Change in the Fiction of J.M. Coetzee,” tags the writer’s ethical responsibility for the *other*, puts it:

After all, the form of engagement in which it results is affective and cannot engage the world of action in terms of action. The paradox, of course, is that, from an ethical perspective, such as obligation is nothing less than infinite. (2000:180).

## Works consulted

Attridge D (2004) J.M. Coetzee and the Ethics of Reading: Literature in the Event. University of Chicago Press, Chicago

Attridge D (Fall 2000) Age of bronze, State of Grace. Music and Dogs in Coetzee’s Disgrace. Novel: A Forum on Fiction

Atwell D (2001) Coetzee and post-apartheid South Africa. J South Afr Stud 27(4):865–867

Coetzee JM (1990) Age of Iron. Secker & Warburg, London

Coetzee JM, Costello E (2003) Eight Lessons. Secker & Warburg, London

Kossew S (2003) The Politics of Shame and Redemption in J.M. Coetzee’s Disgrace. Res Afr Lit 34(2):155–162

Sorensen R (11^th^ Sept. 1999) Review of Disgrace, 11th edn. Brisbane Courier-Mail: p. 7

van Gallagher Zanten S (2002) Truth and reconciliation. The Confessional Mode in South African Literature, NH: Heineman, Portsmouth
